# Presentation of lumbar intramedullary cavernous hemangioma by spindle-shaped hematoma sign on the spinal MRI: a case report

**DOI:** 10.1186/s13256-024-04885-6

**Published:** 2024-11-20

**Authors:** Zhinan Ye, Gaiying Ma, Hongwei Liu

**Affiliations:** 1 Department of Neurology, Municipal Hospital Affiliated to Taizhou University, Taizhou, 318000 Zhejiang China; 2 Department of Neurology, Taiyuan Central Hospital, Shanxi Medical University, No.5, Three Lanes East Road, Taiyuan, 030000 China

**Keywords:** Spinal cord cavernous hemangioma, Spindle-shaped hematoma sign, Magnetic resonance imaging, A case report

## Abstract

**Background:**

Cavernous hemangioma is a congenital insidious disease that can occur in any part of the central nervous system. In clinical practice, cavernous hemangioma is mostly found in the brain and rarely in the spinal cord.

**Case summary:**

This study describes a case of a 34-year-old Chinese man of Han ethnicity with lumbar intramedullary cavernous hemangioma. On admission, lumbar intramedullary hemorrhage was observed in the patient, as well as a spindle-shaped hematoma sign was detected on the spinal magnetic resonance imaging. We suspected that the spinal hemorrhage could be associated with spinal cord cavernous hemangioma. The patient was started on dehydration and glucocorticoid therapy of mannitol on the day of admission and was diagnosed with cavernous hemangioma on the basis of his magnetic resonance imaging presentation and spinal cord histopathology results. However, there was no significant improvement in clinical manifestations following conservative treatment. It was exciting that the patient’s condition improved after the surgical removal of hematomas.

**Conclusion:**

Clinicians should consider lumbar intramedullary cavernous hemangioma in the differential diagnosis of early spinal cord hemorrhage with a spindle-shaped hematoma sign on the spinal magnetic resonance imaging.

## Introduction

In clinical practice, cavernous hemangioma is mostly found in the brain and rarely in the spinal cord. Spinal cord cavernous hemangioma (CH) is usually a common and benign vascular lesion, accounting for 5–12% of all spinal vascular malformations [1,2]. Histopathologically, spinal cord CH is characterized by an abnormally complex network of vasculature with varying sizes and without the involvement of any large artery or vein ^[[3]]^. Clinical presentation of spinal cord CH includes repeated episodes of spinal cord hemorrhages that can lead to a malignant spinal lesion, resulting in a spectrum of neuropathological conditions [3,4]. We herein present a case where the spindle-shaped hematoma sign on the lumbar intramedullary magnetic resonance imaging (MRI) examination aided the diagnosis and identification of spinal cord CH and its radiological features.

## Case presentation

**Fig. 1:**
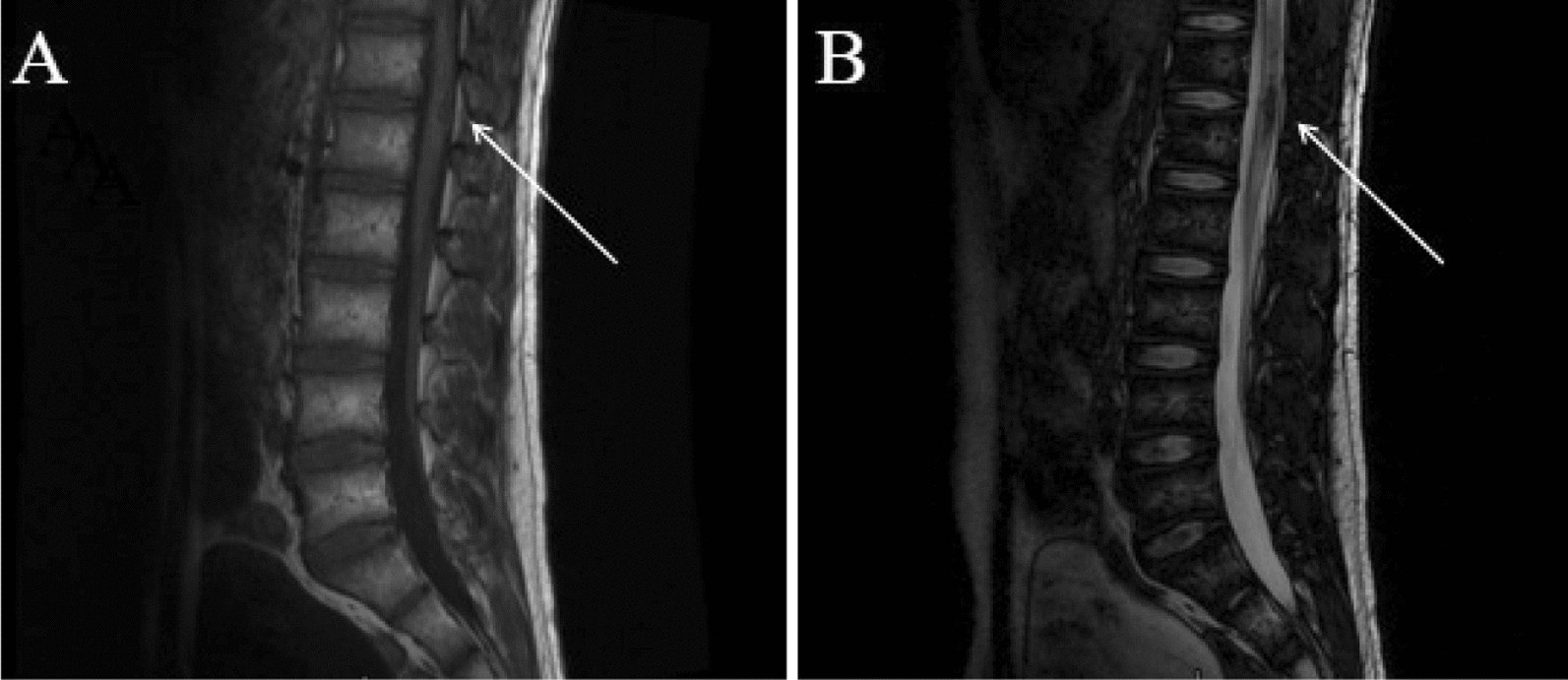
On admission. Sagittal TI-weighted MR (**A**) and T2 weighted MR (**B**) of the lumbar spine. Signal of the spinal cord can be seen extending from T12 to L1 posteriorly along the the fiber tracts (arrows) with associated myelum swelling

**Fig. 2:**
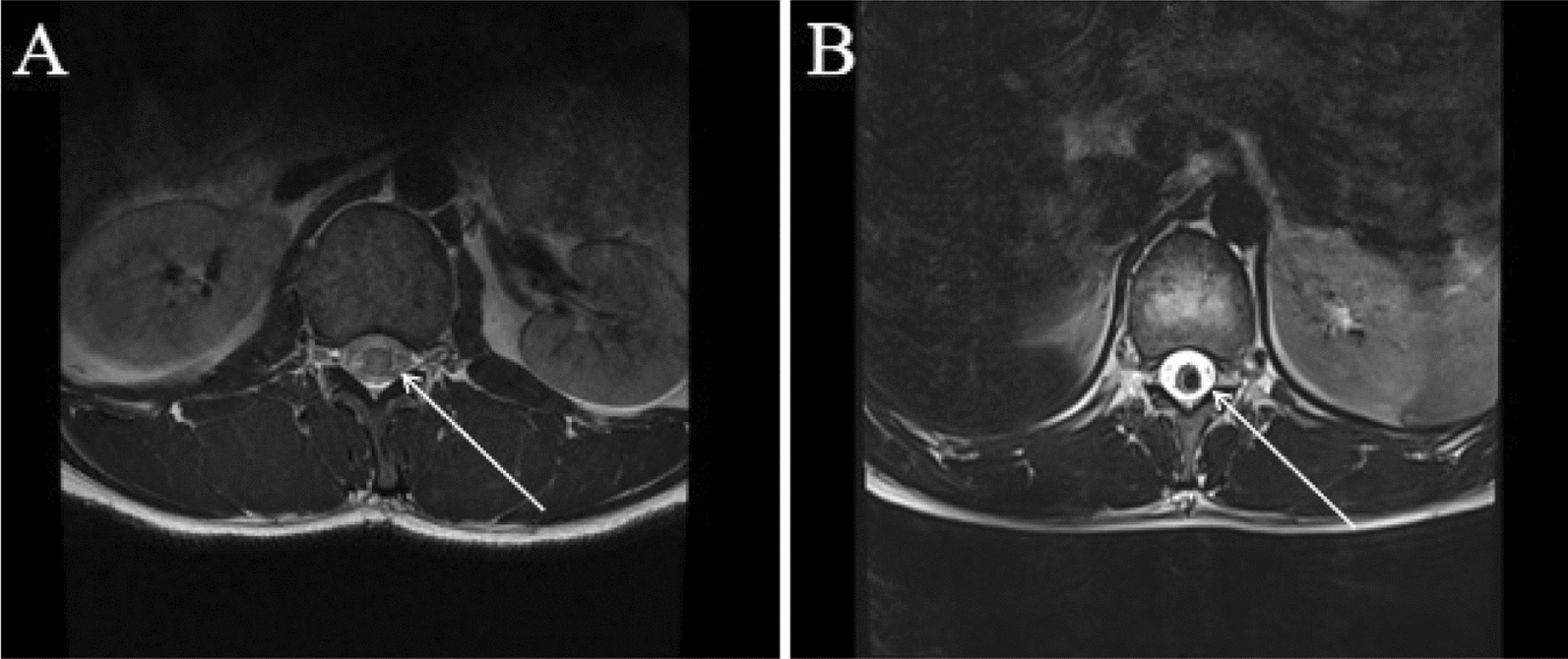
Axial T2 weighted magnetic resonance image (**A** the 2th day of admission and **B** the 10th day of admission) of the lumbar spine with spindle-shaped hematoma sign. Signal of the spinal cord can be seen along the the fiber tracts (arrows)

**Fig. 3:**
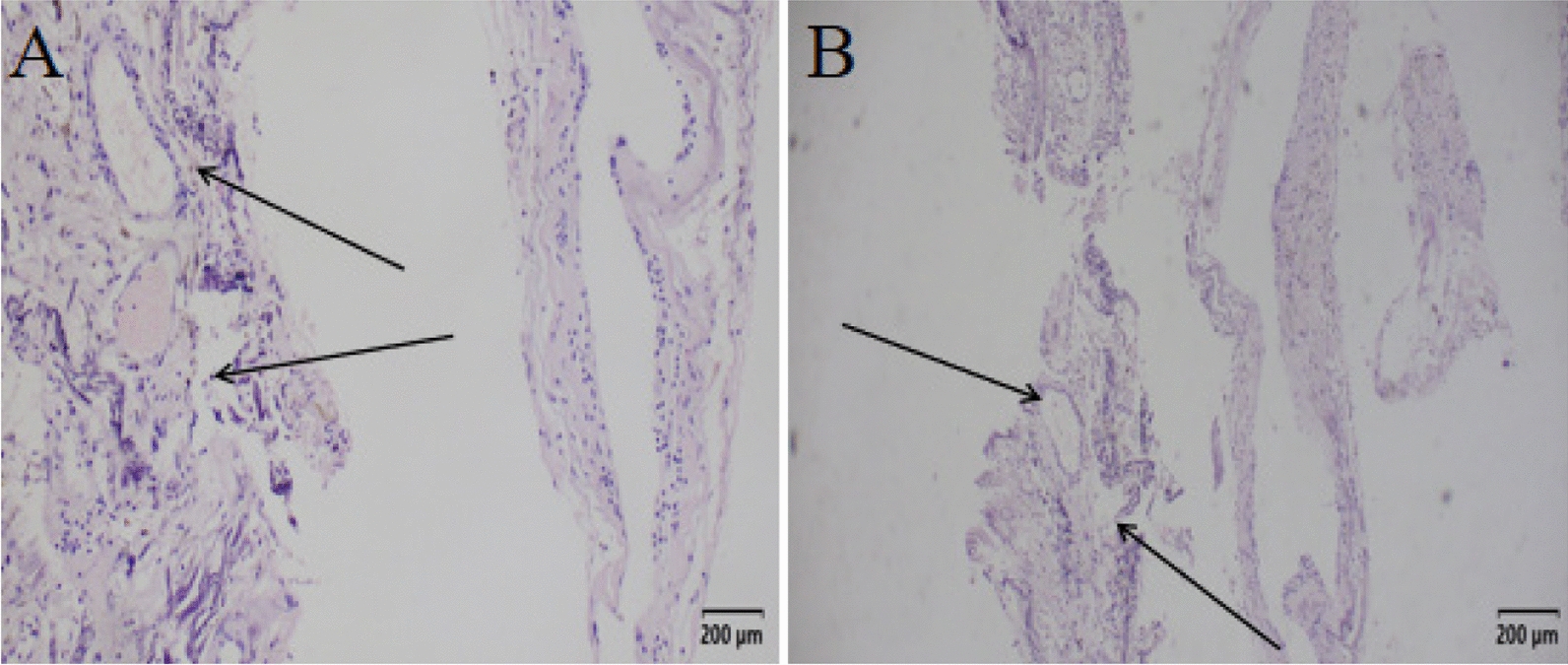
**A** shows a dense network of dilated blood vessels with thin walls and irregularly arranged endothelial cells. **B** offers a closer view of the vessel wall, highlighting thin collagen fibers and well-defined endothelial cells, characteristic of cavernous hemangioma

A 34-year-old Chinese man of Han ethnicity presented with a progressive aggravation of left lower limb weakness and lumbar pain that lasted for 3 days. Before the manifestation of symptoms, the patient was healthy, and had no history of either familial or sporadic bleeding disorder, and was not on oral anticoagulants. On admission, the patient struggled to walk independently and required active support for his physical activities. Additionally, he complained of dysuria. Neurological examination indicated grade 2 muscle strength in the left lower limb, diminished tendon reflexes in the left lower limb relative to normal, and reduced pain and temperature perception in the right lower limb. Routine blood work did not show any abnormal changes. Additionally, metagenomic analysis by next-generation sequencing (NGS) as well as testing for titers for the demyelinating antibodies in the cerebrospinal fluid (CSF) sample could not reveal any pathological changes. Moreover, no brain hemorrhagic lesion was visible on the brain MRI examination. However, an MRI of the lumbar intramedullary showed sagittal short segments of T1 and T2-hypointensity and axial T2-hypointensity with “spindle-shaped hematoma sign,” along the spinal cord tract extending from T12 to L1 (Figs. 1 and 2). At the same time, the MRI of the lumbar intramedullary also displayed swelling in the annular region surrounding the fringe of T2-hypointensity between the T9 and T11. There was no contrast enhancement. On the basis of this evidence, the patient was diagnosed with spinal cord CH and further confirmed by the histopathological examinations (Fig. 3). After identifying the underlying cause, timely decompression surgery was performed using the posterior midline approach to the spinal cord. This procedure successfully alleviated spinal cord compression and utilized the vertebral plate replantation technique to restore spinal stability. Electrophysiological monitoring, including somatosensory and motor evoked potentials, was employed throughout the surgery to refine surgical strategies, anticipate severe spinal injuries, and enhance surgical safety. Additionally, ultrasound was utilized intraoperatively to accurately locate the intramedullary CM, aiding in precise spinal cord incision planning and ensuring complete lesion removal. At 1 week post-surgery, the patient's left lower limb muscle strength improved to grade 3 prior to discharge. At the 6-month follow-up, the patient had regained full ambulatory function, exhibiting a completely normal walking pattern. At 1 year following the surgery, the patient had fully returned to their normal daily activities.

## Discussion

In rare cases, spinal cord hemorrhages may occur owing to certain types of hemangiomas, such as spinal cord CH and intradural arteriovenous malformation. Spinal cord CH are rare lesions. The incidence of spinal cord CH in the real world is currently unknown but reportedly represents 5–12% of all spinal vascular abnormalities [1,2]. Despite a few autopsy reports on partially ante mortem undiagnosed spinal cord CH, the majority of cases are surgically treated lesions. Earlier, its clinical diagnosis was a bit challenging because of the pathognomonic limitations. In recent years, the application of spine MRI has dramatically increased the chances of rapid identification of vertebral hemorrhagic lesions, resulting in the precision diagnosis of spinal cord CH. Notably, the duration of clinical presentation of spinal cord CH symptoms may range from as short as 1 week to 25 years, with a median of 3–4 years, until the diagnosis is confirmed. Regarding the promptness of the symptomatic presentation of spinal cord CH, males are more likely to exhibit the symptoms much earlier, usually in their second or third decades of life, than their female counterparts [1]. However, Aoyagi *et al*., have shown a slightly more female preponderance (1.43:1) in a cohort of 54 patients [5]. The symptom onset age in our patients was similar to that of the peak incidence of cerebral cavernous angiomas.

A study conducted over 12 years has revealed that 41% of spinal cord CH lesions can be found in the cervical region, 57% in the thorax, and only 2% in the lumbar region of the spinal cord [6]. The clinical manifestations and severity of spinal cord CH depend on many etiological factors, such as the location of hemangioma, the amount of bleeding, the degree of spinal cord injury at the involved segment, and the number of hemorrhages, which explains the reason for a wide diversity in the clinical presentations of spinal cord CH. Spinal cord CH can also lead to severe myelopathic symptoms. Spinal cord CH-induced hemorrhage often presents with acute sensory and motor disorders followed by bladder and rectal disturbances. Moreover, neurological symptoms may aggravate owing to hemorrhage-associated tissue damage, which was also consistently observed in our patients at the initial stage. Clinical symptoms of spinal cord hemorrhage tend to follow three patterns: (1) gradual clinical deterioration, (2) recurrent neurological dysfunction with varying degrees of functional recovery during the interictal period, and (3) acute onset with a rapid fast or slow decline over several weeks or months. In a cohort study, Nagoshi N, *et al*. have reported that patients with acute onset, severe neurological damage, and/or gradual aggravation often resulted in poor prognoses [4]. This subset of patients often needed early surgical intervention to relieve spinal cord compression. Spinal MRI showed bleeding signals, which were highly variable in nature and related to the duration of spinal cord CH pathogenesis. Sudden recurrence of symptoms, including recurrent neck and shoulder pain, back pain, or limb dysfunction, were reported in these patients. Interestingly, these symptoms can often be ignored or misdiagnosed. Diagnosis is usually confirmed only after recurrent attacks and gradually aggravating symptoms [4,7]. Typical spinal MRI manifestations are usually “popcorn” or “mulberry” like morphology with mixed signals on the sagittal T1 weighted image (T1WI) and T2WI, low signal annular surrounding area in T2WI, and different degrees of lesion enhancement [8]. Spinal MRI can be used as an important diagnostic approach for spinal cord CH. Notably, the most prominent clinical feature of spinal cord CH in our patients was the spindle-shaped hematoma sign, which was consistent with early lumbar intramedullary hemorrhages on T2WI. Since previous studies have not investigated the clinical characteristics of spindle-shaped hematoma signs, it remains unknown whether this is an early indication of spinal cord hemorrhage. More research is needed to confirm this imaging characteristic in the future. The posterior spinal approach presents several distinct advantages. It enables precise dural suturing while obviating the need for thoracotomy in the thoracic region, thereby mitigating the associated risks to the heart, lungs, major vasculature, and mediastinal structures. When compared with total or full laminectomy, the semi-laminectomy technique demonstrates superior surgical outcomes, including a reduction in iatrogenic injury, diminished posterior traction, and improved preservation of spinal stability.

Although spinal cord CH is not a real tumor, it has the characteristics of repeated bleeding and slow growth. Additionally, it is located in the spinal cord and has a narrow compensatory space, which often leads to progressive spinal cord degeneration, resulting in paraplegia or more severe motor deficits. In their study on a cohort of 71 patients with untreated spinal cord cavernous malformations, Santos *et al*. found an increased risk for repeated hemorrhages in cavernous malformations of the spinal cord compared to that in cerebral cavernous malformations [8]. Reportedly, neurological functions significantly deteriorated after the second bleeding, and the possibility of recurrent intramedullary hemorrhage increased drastically after the first hemorrhage. Therefore, there is debate on whether it is necessary to exhaustively remove the lesion focus or apply conservative treatments. A systemic review following the preferred reporting items for systematic reviews and meta-analyses guidelines has revealed that surgical removal of multiple lesions within 3 months of symptom onset can significantly improve the neurological condition in 36.9% of patients, stabilize the condition in 55.8% of patients, or deteriorate the condition in 7.3% of patients [9]. In another study involving a conservative cohort, 21.7% of patients exhibited improvement, 69.6% remained stable, and 8.7% deteriorated [10]. Prospective studies in a larger cohort would be needed to confirm the benefits of conservative treatment in patients with symptomatic spinal cord CH.

## Conclusion

Although there is no typical “popcorn” or “mulberry” morphological sign in spinal MRI, the diagnostic information provided by the spindle-shaped hematoma sign on MRI can be valuable for identifying the disease characteristics and etiopathology of early symptomatic spinal cord hemorrhage, which could help to guide the disease management in patients with spinal cord CH.

## Data Availability

Not applicable.
